# Mamma Mia – A randomized controlled trial of an internet-based intervention for perinatal depression

**DOI:** 10.1017/S0033291718002544

**Published:** 2018-09-07

**Authors:** Silje Marie Haga, Filip Drozd, Carina Lisøy, Tore Wentzel-Larsen, Kari Slinning

**Affiliations:** 1 Department for Infant Mental Health, Regional Centre for Child and Adolescent Mental Health, Eastern and Southern Norway, Oslo, Norway; 2 Norwegian Center for Violence and Traumatic Stress Studies, Oslo, Norway; 3 Department of Psychology, University of Oslo, Oslo, Norway

**Keywords:** Internet intervention, linear mixed effects models, perinatal depression, randomized controlled trial, universal prevention

## Abstract

**Background:**

Studies suggest that 10–15% of perinatal women experience depressive symptoms. Due to the risks, problems with detection, and barriers to treatment, effective universal preventive interventions are needed. The aim of this study was to assess the effectiveness of an automated internet intervention (‘Mamma Mia’) on perinatal depressive symptoms. Mamma Mia is tailored specifically to the perinatal phase and targets risk and protective factors for perinatal depressive symptoms.

**Methods:**

A total of 1342 pregnant women were randomized to an intervention (‘Mamma Mia’) and control group. Data were collected at gestational week (gw) 21–25, gw37, 6 weeks after birth, and 3 and 6 months after birth. We investigated whether (1) the intervention group displayed lower levels of depressive symptoms compared with the control group, (2) the effect of Mamma Mia changed over time, (3) the effect on depressive symptoms was moderated by baseline depressive symptoms, previous depression, and parity, and (4) this moderation changed by time. Finally, we examined if the prevalence of mothers with possible depression [i.e. Edinburgh Postnatal Depression Scale (EPDS)-score ⩾10] differed between the intervention and control group.

**Results:**

Participants in the Mamma Mia group displayed less depressive symptoms than participants in the control group during follow-up [*F*(1) = 7.03, *p* = 0.008]. There were indications that the effect of Mamma Mia was moderated by EPDS score at baseline. The prevalence of women with EPDS-score ⩾10 was lower in the Mamma Mia group at all follow-up measurements.

**Conclusions:**

The study demonstrated the effects of the automated web-based universal intervention Mamma Mia on perinatal depressive symptoms.

## Introduction

The perinatal period represents a time signified by much emotional turmoil for women, and studies suggest that 10–15% of women experience moderate to high levels of depressive symptoms during this period (O'Hara and Swain, 1996; Eberhard-Gran *et al*., 2004; Gavin *et al*., 2005). Factors associated with increased risk of perinatal depression include a history of depression, stressful life events, young age and other demographic variables (Robertson *et al*., 2004; Vesga-López *et al*., 2008; Lancaster *et al*., 2010; Silverman *et al*., 2017). A majority of studies emphasize the postpartum period as a particularly vulnerable time during which the woman is at increased risk for mental disorders (Munk-Olsen *et al*., 2006; Vesga-Lopez *et al*., 2008; O'Hara and McCabe, 2013). More recent studies, however, highlight how women seem to be at equal risk of developing depression during pregnancy (Melville *et al*., 2010), and approximately 50% of depressive episodes after birth, start during pregnancy (American Psychiatric Association, 2013). This underscores the importance of early intervention, as the chronicity of depressive symptoms is of particular concern for the consequences of depression (Sohr-Preston and Scaramella, 2006). Perinatal depression carries a high personal cost for both the woman and her family (Lovestone and Kumar, 1993; Lovejoy *et al*., 2000; Goodman *et al*., 2011) while all too often escaping detection and treatment (Bågedahl-Strindlund and Monsen Børjesson, 1998).

Although effective psychological treatments for perinatal depression exist (Stuart-Parrigon, and Stuart, 2014; Stephens, 2016), there are several complicating factors that impede women's ability to receive appropriate treatment, such as limited access to treatment (Payne and Myhr, 2010; WHO, 2015), difficulties in attending therapy during usual business hours, struggles with geographical location (Overland *et al*., 2007), transportation and childcare (Goodman, 2009). Additionally, many women are reluctant to seek treatment due to a perceived stigma (Beck, 2001; O'Mahen and Flynn, 2008; Goodman, 2009). Some women even fear that their children will be ‘taken away’ if health providers discover that they suffer from perinatal depression (Dennis and Chung-Lee, 2006). Furthermore, many new mothers are unfamiliar with what constitute depressive symptoms, and do not recognize that they suffered from perinatal depression until it was over. Consequently, many people suffer ‘in silence’. The many barriers associated with face-to-face treatments clearly underscore a need for innovative approaches to public health prevention and promotion.

Internet-delivered interventions represent an innovative approach that circumvents many of the obstacles encountered in the dissemination of face-to-face treatment (Kohn *et al*., 2004). Internet interventions allow anonymity, which reduces a sense of stigma and patients’ fear of seeking help (Dennis and Chung-Lee, 2006). In turn, such interventions can more readily reach the many people that suffer ‘in silence’. Indeed, many women with perinatal depression express an interest in internet interventions and report that they would use the internet to learn coping strategies for depressive symptoms (Maloni *et al*., 2013). Because of the scalability, internet-based interventions are likely cost-effective, which makes them especially advantageous for disorders characterized by a combination of high prevalence and low treatment-seeking rates.

Recent studies have demonstrated the acceptability and feasibility of internet interventions for perinatal depression (Danaher *et al*., 2012; Haga *et al*., 2013; Salonen, 2014; Logsdon *et al*., 2014). Findings from two recent systematic reviews on web-based interventions for perinatal mental health are encouraging and suggest they may be effective in reducing perinatal depression (Ashford *et al*., 2016; Lee *et al*., 2016). However, there is still an overall need for high-quality randomized controlled trials (RCTs) to assess the effects of web-based interventions on perinatal depression (Lee *et al*., 2016). Interestingly, the reviews address how remarkably few of the existing interventions are tailored specifically to pregnancy and the postpartum period, which is curious considering how perinatal women face changes, difficulties, and mental health issues that are quite specific to this period (Ashford *et al*., 2016). Interventions that target perinatal specific themes may increase perceived relevance and acceptability, which, in turn, may lower attrition rates (O'Mahen *et al*., 2012). The current intervention, Mamma Mia, was tailored specifically to the perinatal phase and targets risk and protective factors for perinatal depressive symptoms such as attachment, couple satisfaction, social support, and subjective well-being. It was developed to address the high prevalence rates of perinatal depressive symptoms and the need for preventive interventions. Our study aims to contribute to the understanding of the effectiveness of fully automated web-based preventive interventions for perinatal depression.

### Aim of the study

The objective of the current study was to test the effectiveness of a universal preventive intervention for perinatal depressive symptoms (Mamma Mia) on depressive symptoms from pregnancy to 6 months after birth. Our main hypothesis was that the Mamma Mia group would display lower levels of depressive symptoms compared with the control group as measured by the Edinburgh Postnatal Depression Scale (EPDS) (Cox *et al*., 1987). Second, we investigated whether the effect of Mamma Mia changed over time. Third, we examined if the effect of Mamma Mia on depressive symptoms was moderated by baseline depressive symptoms, an indication of previously experienced depression, and parity, and whether this moderation changed over time. Finally, we examined if the prevalence of mothers with at least a probable minor depression (i.e. EPDS-score ⩾10) differed between the intervention and control group.

## Method

### Study design and recruitment

This was a two-armed RCT registered at http://www.controlled-trials.com (ISRCTN91808706). Participants were randomly assigned (ratio: 1:1) to one of two conditions: the intervention group (i.e. usual perinatal care and the web-based program Mamma Mia) or control group [i.e. usual perinatal care (Norwegian Directorate of Health, 2005, 2013)]. The overall goal with the well-baby clinic that offers perinatal care is to promote physical and mental health and prevent injuries and disease, and ensure a continuity of care for its users (Norwegian Directorate of Health, 2004). According to guidelines for prenatal care, all women are offered eight consultations throughout the pregnancy, and midwives are to identify women in need of extended care and offer additional consultations (Norwegian Directorate of Health, 2005). During the first 6 months following birth, the woman and her child should be offered six consultations. Although the child's development is of primary concern during these consultations, the psychological well-being of the mother is also addressed. Pregnant women across Norway were invited to take part in the study between December 2013 and February 2015. Participants were recruited at well-baby clinics across the country during routine prenatal care and via hospitals in eastern Norway during regular ultrasound [i.e. gestational week (gw)18–20]. Eligible participants had to be pregnant (up until gw25), at least 18 years of age, able to read and write Norwegian, have access to the internet and have an electronic mailing account.

### The intervention – Mamma Mia

Participants in the intervention received Mamma Mia; a universal preventive intervention for perinatal depressive symptoms. It is a fully automated internet-based program available free of charge. The intervention comprises three phases. The first phase consists of 11 sessions beginning in the second trimester in gw 21–25 and ends in gw37. The second phase starts when the infant is 2–3-weeks-old, and lasts for 6 weeks, with three sessions per week. The final phase consists of 10 sessions over an 18-week period. In total, the intervention consists of 44 sessions over a period of 11.5 months. All sessions include themes specific to the perinatal period. Mamma Mia applies a tunneled design to guide the woman through the program in a step-by-step fashion in accordance with the psychological preparations of becoming a mother. The intervention is delivered by email and interactive websites, combining text, pictures, prerecorded audio files, and user input. Each session is designed to take about 10 min and must be completed before users can access the next session. This is done to ensure that relevant information has been reviewed and to create continuity and a narrative in the program (see Drozd *et al*., 2015 for a comprehensive description of Mamma Mia). A recent study demonstrates the feasibility and acceptability of Mamma Mia (Haga *et al*., 2013). For a demonstration of Mamma Mia, see: http://smarturl.it/psych_med.

### Data collection and randomization

Data was gathered by means of internet-based surveys. First, participants reported on demographic information (incl. age, marital status, parity, education, and estimated due date). Based on the reported due date, it was calculated when participants were between gw21–25, and that was when they received their baseline questionnaire assessing depressive symptoms. This was done to ensure similarity in terms of when depressive symptoms were assessed. Upon completing the baseline assessment, an automated, unrestricted randomization procedure was carried out. Thus, each participant had an equal probability of being assigned to each of the two groups, resulting in 678 and 664 eligible participants being randomly assigned to the intervention and control group, respectively. Depressive symptoms were assessed twice during pregnancy; at baseline between gw21 and 25 and at gw37. After birth, depressive symptoms were assessed at 6 weeks, 3 and 6 months postpartum.

Depressive symptoms were measured by EPDS (Cox *et al*., 1987), which is a 10-item self-report instrument that assesses depressive symptoms during the last 7 days. It has been validated for use during pregnancy (Bunevicius *et al*., 2009; Bergink *et al*., 2011), and postpartum (Eberhard-Gran *et al*., 2001*a*). Items are rated on a 4-point scale from 0 to 3 to produce a summative score ranging from 0 to 30, with higher scores indicating elevated risk for perinatal depression. Cox *et al*. (1987) recommend a cut-off score of EPDS ⩾10 to detect possible depression. The present study used the validated Norwegian translation of the EPDS (Eberhard-Gran *et al*., 2001*b*). Continuous EPDS-scores were used in main analyses, as recommended for the EPDS in population research (Green, 2005), while a cut-off score of ⩾10 was used to assess group differences in prevalence rates of possible depression across time.

### Statistical analysis

Baseline differences between groups were merely examined by descriptive information in line with recommendations in the CONSORT guidelines. Logistic regression analyses with group assignment as an independent variable were done at each measurement time to assess drop-out after baseline, unadjusted and adjusted for age, first language, parity, education, previous depression, and EPDS at baseline. A series of linear mixed effects (LME) models were estimated for the time development of EPDS-scores after randomization. Time was included as a categorical independent variable with four categories (gw37, 6 weeks, 3 and 6 months postpartum), and treatment assignment was included as a dichotomous independent variable (control or Mamma Mia). Mixed-effects models are commonly used for repeated measures data (Pinheiro and Bates, 2012) as they do not require balanced data and give valid results under the less restrictive missing at random assumption. LME models handle repeated measure data in a long format and are therefore especially advantageous in longitudinal studies with missing data, as all data available is used and no cases are deleted. All models included a random intercept. For fixed effects, the first model was by group and time only. The second model included a group by time interaction to test for differences in intervention effects during follow-up. The third model also included interactions of group by parity, previous depression and depressive symptoms at baseline, and the final fourth model included third-order interactions between the group, time, and these background characteristics. It should be noted that EPDS-scores at baseline were not included in the dependent variable because when randomization is successful, group differences at baseline are random.

In supplementary analyses of the prevalence of depression over time after randomization, we performed logistic regression analyses for repeated measurements by means of generalized estimating equations (GEE). The binary outcome was EPDS-scores <10 (coded as 0) and EPDS-scores ⩾10 (coded as 1). In addition, we performed separate logistic regressions at each time point, adjusted for a baseline from gw37 to 6 months postpartum. The significance level was set to 0.05, but in line with the recommendations of the American Statistical Association, this boundary was not used strictly (Wasserstein and Lazar, 2016). All tests were two-tailed. SPSS version 23 (IBM SPSS, Armonk, NY, USA) was used for descriptive analyses. The R (The R Foundation for Statistical Computing, Vienna, Austria) package nlme was employed for the mixed effects analyses and the GEE analyses employed the R package gee.

## Results

**Fig. 1.**
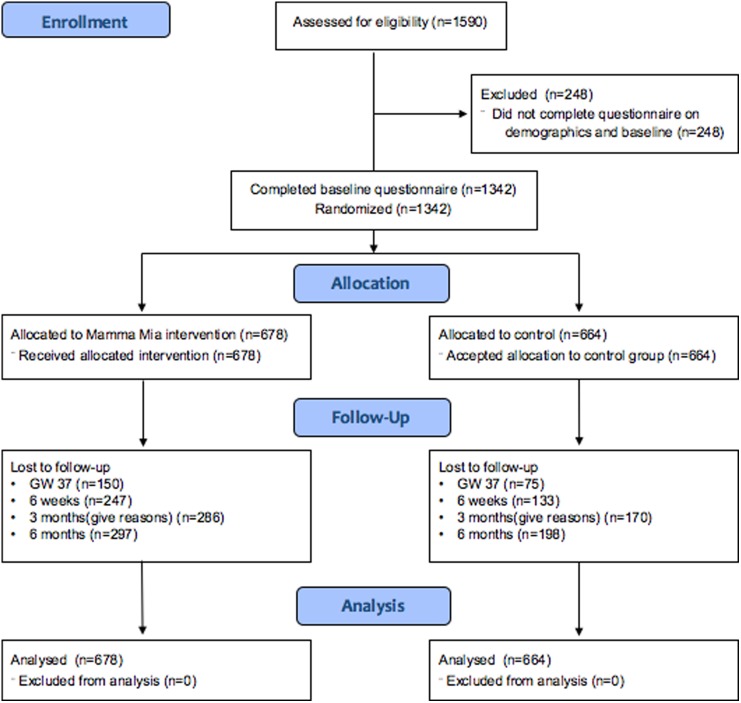
Participant flowchart.

The flow of participants is illustrated in Fig. 1. In total, 678 participants were randomly assigned to the Mamma Mia group, and 644 were randomly assigned to the control group.

The number of respondents in the total sample was 1342 at baseline, 1117 (83.2%) at gw37, and 962 (71.7%), 886 (66.0%), 847 (63.1%) at 6 weeks, 3 and 6 months postpartum, respectively.

Demographics are presented in Table 1. Mean maternal age was comparable with that of the average age for all births in Norway, which is 30.8 years (Statistics Norway, 2017*b*). In terms of education and parity, more than half of the mothers reported having higher education (i.e. ⩾4–5 years in college or university) and being primipara. This may indicate that the level of education was substantially higher than the 8.7% of women with higher education in the general population (Statistics Norway, 2017*a*), although the level of education among pregnant women in Norway is unknown. Using mothers’ first language as a proxy for ethnicity, the proportion of mothers with non-Scandinavian language was 7.7%, while the proportion of births given by mothers born outside of Norway was 27% in 2014 (The Norwegian Insitute of Public Health, 2015). The skewness in ethnicity may, however, be explained by the implicit inclusion criterion; the ability to read and understand Norwegian to participate in Mamma Mia.

**Table 1.** Participant characteristics

Characteristic	Mamma Mia (*n* = 678)	Control (*n* = 664)
Age (years) mean (s.d.)	31.0 (4.6)	31.1 (4.5)
Education *n* (*%*)		
⩽ high school	100 (14.7)	107 (16.1)
1–3 years college or university	189 (27.9)	183 (27.6)
⩾4–5 years college or university	389 (57.4)	374 (56.3)
First language *n* (%)		
Scandinavian	630 (92.9)	609 (91.7)
Non-Scandinavian	48 (7.1)	55 (8.3)
No. of previous children *n* (%)		
No previous children	393 (58.0)	382 (57.5)
⩾1 children	285 (42.0)	282 (42.5)

Table 2 shows levels of depressive symptoms in the Mamma Mia and control group over time. It also shows the percentage of participants who scored ⩾10 on the EPDS, which is an indication of possible depression. As can be seen, levels of depressive symptoms were highest at baseline in both groups, and the greatest reduction in symptoms occurred from baseline (gw21–25) to gw37. Noticeably, the proportion of women scoring above the cut-off at baseline was just over 23% in both groups. This is a higher proportion than what recent studies have found in Norway (Eberhard-Gran *et al*., 2002; Dørheim *et al*., 2009; Glavin *et al*., 2010; Haga *et al*., 2012, 2017), indicating that perhaps women with higher levels of depressive symptoms than the rest of the perinatal population have signed up for the study. This is not surprising considering that the participants were self-selected, and the study revolved around an intervention for perinatal depression.

**Table 2.** Means, standard deviations, and number of women scoring above the cut-off for EPDS over time (*N* = 1 342)

	Mamma Mia (*n* = 678)			Control (*n* = 664)		
	*n* (%)	EPDS (mean, s.d.)	EPDS ⩾10 (valid *n*, %)	*n* (%)	EPD (mean, s.d.)	EPDS ⩾10 (valid *n*, %)
Baseline	678 (100.0)	6.5, 4.5	160 (23.6)	664 (100.0)	6.2, 4.4	156 (23.5)
gw37	528 (77.9)	5.2, 4.0	72 (13.6)	589 (88.7)	5.8, 4.3	104 (17.7)
6 weeks	431 (63.6)	5.2, 4.1	60 (13.9)	531 (80.0)	5.8, 4.2	94 (17.7)
3 months	392 (57.8)	4.1, 4.0	37 (9.4)	494 (74.4)	4.5, 4.2	63 (12.8)
6 months	381 (56.2)	4.0, 4.0	33 (8.7)	466 (70.2)	4.4, 4.3	55 (11.8)

### Drop-out and missing data

At all measurement times, drop-out was greater in the intervention group [odds ratio (OR) = 0.43–0.55, all *p*s <0.001 both in adjusted and unadjusted analysis]. There was little difference when comparing the adjusted and unadjusted analyses. For the whole sample, later dropout was predicted by a higher score on baseline EPDS (all *p*s ⩽0.047) and lower level of education (all *p*s ⩽0.004).

### Adherence to the intervention

Adherence to the intervention refers to the completion of sessions. A total of 678 participants received the Mamma Mia intervention. Of these, 226 (33%) completed all 44 sessions. As many as 345 (51%) completed 36 or more sessions (>80% of the intervention), and merely 41 (6%) participants did not initiate using the program. The completion rate is high considering that Mamma Mia is a comprehensive, unguided, and fully automated prevention program that is delivered at a time that is already quite hectic.

### Symptom reduction

**Fig. 2.**
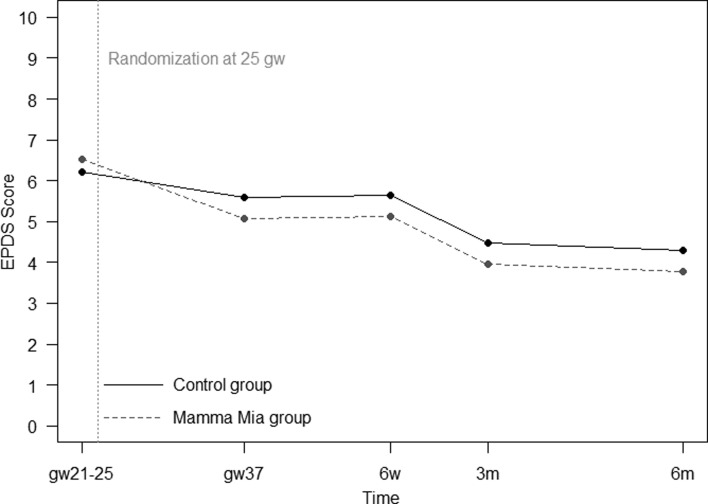
Mamma Mia and control group trajectories of depressive symptoms. Numbers at baseline are means, while numbers during follow-up are model-based estimates.

Our main hypothesis was that the level of depressive symptoms would differ between treatment and control group. The first model, by treatment assignment and time, showed a significant effect of Mamma Mia on depressive symptoms [*F* (1) = 7.03, *p* = 0.008] (see Fig. 2).

### Effect on depressive symptoms over time

In the second model, there were no significant differences in the rate of change between the Mamma Mia and control group over time [*F* (3) = 1.02, *p* = 0.384 for the time by group interaction]. This means that the intervention group had lower levels of depressive symptoms compared with the control group, but both groups seemed to follow a similar trajectory in depressive symptoms. As seen in Table 3, participants in the Mamma Mia group had significantly lower symptoms than the control group at gw37 and 6 weeks postpartum. Group differences were not statistically significant at 3 and 6 months postpartum.

**Table 3.** Contrasts between the Mamma Mia and control group at different time points (*N* = 1117)

	Model 2 (Conditional)			
Time	Contrast	Lower 95% bound	Upper 95% bound	*p* value
gw37	−0.65	−1.13	−0.17	0.008
6 weeks	−0.56	−1.07	−0.05	0.031
3 months	−0.31	−0.84	0.21	0.241
6 months	−0.25	−0.78	0.28	0.358

### Testing moderating variables – over time

In the third model, only EPDS-scores at baseline moderated the effect of group (*p* = 0.051). The group difference was somewhat more pronounced for higher values of baseline EPDS (data not shown). All other interactions were clearly non-significant (all *p*s ⩾0.317). In the final model, there was no indication of any third-order interactions (all *p*s ⩾0.133).

In sum, results from the mixed effects models indicate that participants in the Mamma Mia group scored lower on depressive symptoms than participants in the control group on EPDS during follow-up (gw37–6 months). The differences between the groups were significant at gw37 and 6 weeks. There were indications that the effect of Mamma Mia was moderated by EPDS score at baseline; indicating that a higher initial EPDS-score yielded a greater effect of Mamma Mia.

### Prevalence of possible depression

**Fig. 3.**
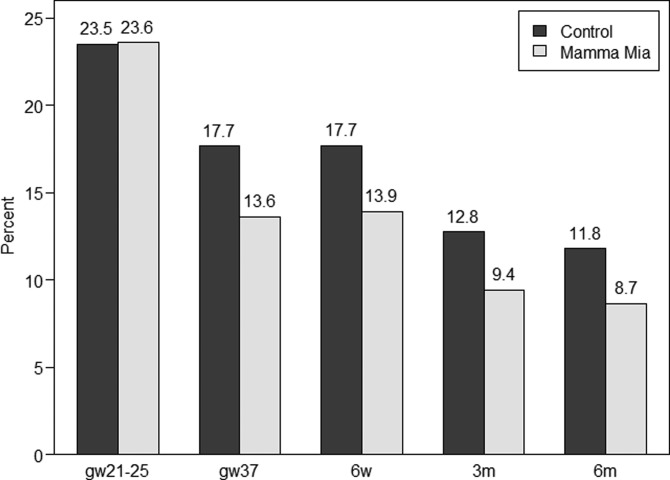
Percentages of women with EPDS-scores ⩾10 in the Mamma Mia and control group across time.

Finally, we examined if the prevalence of women with a possible minor depression (i.e. EPDS-score ⩾10) differed between the intervention and control group. Figure 3 below presents a histogram showing the percentage of women with EPDS-scores ⩾10 across time in the Mamma Mia and control group, respectively. These results indicate a 21.5–26.6% reduction in the prevalence of possible depression in the Mamma Mia group compared with the control group across time. The prevalence of women who scored ⩾10 on the EPDS was lower in the Mamma Mia group from gw37 and onwards (GEE adjusted for time and baseline, OR 0.75, 95% CI 0.58–0.96, *p* = 0.024). This difference was fairly stable over time (ORs between 0.71 and 0.78, see Table 4).

**Table 4.** Results from separate logistic regression analyses comparing Mamma Mia to controls at each time point using EPDS ⩾10 as an outcome

	gw21–25	gw37	6 weeks	3 months	6 months
OR (95% CI)	1.01	0.74	0.78	0.72	0.71
	(0.78–1.29)	(0.52–1.04)	(0.55–1.13)	(0.46–1.12)	(0.45–1.13)
*p* value	0.964	0.082	0.191	0.141	0.149

Estimates from gw37 to 6 months are adjusted for baseline EPDS.

## Discussion

The focus of this study was to evaluate the effectiveness of an internet-based program (‘Mamma Mia’) on depressive symptoms from pregnancy to 6 months postpartum. Results indicated that the reduction in mean depressive symptoms in the intervention group compared with the control group was small but significant. In fact, participants who received Mamma Mia displayed less depressive symptoms than participants in the control group on all measurements occasions after baseline. This difference was statistically significant at gw37 and 6 weeks postpartum. Although group differences were no longer statistically significant at 3 and 6 months postpartum, participants in the intervention group still had lower levels of depressive symptoms than the control group. As symptom severity is at its highest towards the end of pregnancy and early postpartum period (Cox *et al*., 1993; Eberhard-Gran *et al*., 2003; Gaynes *et al*., 2005; Munk-Olsen *et al*., 2006), and typically remits over time (Heron *et al*., 2004), it is not surprising that Mamma Mia was most effective during the third trimester and early postpartum period. Importantly, as the end of pregnancy (Eberhard-Gran *et al*., 2004) and first weeks after birth are particularly vulnerable time periods, it is reassuring that Mamma Mia is most effective at precisely those times.

Although Mamma Mia is a universal intervention, analyses were undertaken to assess whether Mamma Mia was more effective given certain background characteristics. Only EPDS-scores at baseline emerged as a moderator, indicating that a higher initial EPDS score yielded a greater effect of Mamma Mia. This finding is clinically meaningful as people with higher levels of distress have greater potential for improvement (Dennis, 2005).

### The clinical implications of Mamma Mia for the individual and the population

Although the effect on the mean level of EPDS-scores was modest, is important to consider the clinical implications of Mamma Mia for the individual. Interesting research has investigated whether ‘a touch of depression matters’ (Brenner, 1985; Rose, 2008). What Brenner (1985) found was that the likelihood of needing health care support increased with every increase on a scale measuring depressive symptoms, regardless of whether the person was over or below the cut-off score. This finding implies that even the mildest subclinical degree of depression is problematic for the individual and associated with impaired functioning, suggesting that even a minor reduction in depressive symptoms over time will matter for a new mother and her baby. Because all levels of symptoms matter to the people involved, preventive medicine ought to target the whole spectrum of illness and health. What is more, ‘the mild symptom can be the father of the severe’ (Rose, 2008).

In a population, the mean level of symptoms is related to the prevalence of a disorder (Anderson *et al*., 1993; Whittington and Huppert, 1996). Universal interventions that can reduce mean symptoms will thus, in turn, reduce the prevalence of a disorder. Rose (2008) underscored this argument by using an analogy of how the ‘visible part of the iceberg (prevalence) is a function of its total mass (the population average)’ (Rose, 2008). In the current study, analyses supported this analogy by showing that a small mean sample reduction in depression was also accompanied by a reduced proportion of women with an EPDS-score ⩾10 in the Mamma Mia group. Indeed, EPDS-score of ⩾10 prevalence rates were reduced between 21.5 and 26.6% in the Mamma Mia group compared with the controls, across time. These are promising results indicating that Mamma Mia may prevent at least minor to moderate depression. Due to the substantial barriers to care once women become ill, Mamma Mia holds a considerable potential for clinical effectiveness.

### Study limitations

Current drop-out rates fall within the typical range reported on internet-based interventions (Christensen *et al*., 2009), but is nevertheless a limitation in this study. The drop-out was greater in the intervention group than the control group and missing data analysis showed that participants with less formal education and those with higher EPDS-scores at baseline were more likely to drop out. Therefore, EPDS baseline score and educational level were used as covariates in the regression models as a deliberate effort to preclude systematic differences between drop-outs and completers. Nevertheless, both from a methodological and a prevention standpoint, it is unfortunate that participants with more depressive symptomatology were more likely to drop out. Participant drop-out may also have affected the power to detect differences, particularly at later measurement occasions because drop-out became more pronounced over time.

While we used continuous EPDS-scores in our main analyses, an EPDS-score ⩾10 was used as an indicator of possible depression. However, while the EPDS-scores are indicative of depressive symptomatology, scores do not confirm or refute the presence of depression (Cox and Holden, 2003). Cases were not confirmed with clinical assessment. The mean EPDS-scores in both groups at baseline, as well as the proportion of women with an EPDS-score ⩾10, were higher than found in recent Norwegian studies (Dørheim, 2009; Glavin *et al*., 2010; Haga *et al*., 2012; Haga *et al*., 2017). This is likely due to the self-selection of participants and that women with more depressive symptomatology may have been attracted to an intervention that targets depression. Thus, a limitation of the present study is the potential for sampling bias from not achieving a full inclusion of the intended population.

Finally, as in any effectiveness trial, a mix of good and poor responders will have been recruited, so the average therapeutic response may have been mitigated.

### Future recommendations and studies

Research demonstrates how few of the existing interventions are tailored to the perinatal period, which is surprising as tailored interventions are likely perceived as more relevant and acceptable, and, in turn, associated with lower attrition rates. Moreover, perinatal care that is tailored to the woman's needs has been found to reduce the prevalence rate of perinatal depression (MacArthur *et al*., 2002). Mamma Mia is a comprehensive intervention that addresses a wide range of themes that preoccupy perinatal women, ranging from attachment, breastfeeding, regulatory capacity in infants to partner relationship, all of which comprise important health determinants for perinatal depression. A key challenge with such a comprehensive program, however, is the ‘black box’ phenomenon. That is, it is not clear which components are effective for whom and what minimum dosage of the intervention is required to achieve the effect. Moreover, the intervention might be too comprehensive for those most at risk. Future studies should attempt to link the use of intervention components and their effects and determine their dose-response relationships.

Although the current study took place in Norway, the content of Mamma Mia was largely developed based on international studies addressing risk and protective factors for perinatal depression. Moreover, the national clinical guidelines for perinatal care in Norway are in line with World Health Organization recommendations (2015) and practices are comparable with those in Britain (National Institute for Health and Excellence, 2014) and the other Scandinavian countries (Danish Health Authority, 2013; The Swedish Association of Midwives, 2016). Thus, there are reasons to assume that the current results are generalizable to other Western cultures. This should be addressed in future studies.

Recent research shows that internet-based programs *in combination with guidance* from health personnel for treating perinatal depression may produce effects equivalent to those of face-to-face interventions (Cuijpers *et al*., 2010). Therefore, guidelines for the clinical use of Mamma Mia (i.e. with added guidance) and implementation in well-baby clinics have been developed (Drozd *et al*., 2017), designed to fit with existing national guidelines for pre- and postnatal care for well-baby clinics (Norwegian Directorate of Health, 2005, 2013). With a few case-finding questions health personnel could identify women at risk of depression who should be offered the guided program, while the unguided version of Mamma Mia, as tested in this study, may continue to be disseminated as universal prevention. A future study should, however, investigate whether adding guidance by well-baby personnel enhances the effects Mamma Mia. However, until then, conditions are in place for primary care to make the unguided internet-based program available as universal prevention to perinatal women.

## Conclusion

Due to the prevalence, substantial risks, problems with detection of perinatal depression, as well as the substantial barriers to care once women become ill, an effective universal intervention holds substantial potential for clinical effectiveness. The present study demonstrated promising effects of Mamma Mia, and it is readily available for perinatal women at no cost. Primary health services are in a position to relay information about preventive programs for perinatal depression, and Mamma Mia has the potential to aid health care professionals in preventing depressive symptoms in the perinatal population.

## Ethics

The authors assert that all procedures contributing to this work comply with the ethical standards of the relevant national and institutional committees on human experimentation and with the Helsinki Declaration of 1975, as revised in 2008. The participants were regularly screened for depressive symptoms, and those scoring above a given threshold were provided feedback about where they could receive support and help (beyond what the program could offer). Power analyses were conducted so as not to include (and burden) any more participants than necessary. The trial was approved by the Regional Ethics Committee, Norway, South East (project number: 2012/1716).
